# Structural and solvent control over activation parameters for a pair of retro Diels-Alder reactions

**DOI:** 10.1038/s41598-019-54156-4

**Published:** 2019-12-04

**Authors:** Andrea L. Widstrom, Benjamin J. Lear

**Affiliations:** Department of Chemistry, The Pennsylvania State University, University Park, PA USA

**Keywords:** Organic chemistry, Reaction kinetics and dynamics

## Abstract

We report the temperature dependent NMR of two Diels-Alder adducts of furan: one formed with maleic anhydride and the other with N-methylmaleimide. These adducts are the products of so-called ‘click’ reactions, widely valued for providing simple, reliable, and robust reactivity. Under our experimental conditions, these adducts undergo a retro Diels-Alder reaction and we use our temperature dependent NMR to determine the rates of these reactions at multiple temperatures—ultimately providing estimates of the activation parameters for the reversion. We repeat these measurements in three solvents. We find that, in all solvents, the barrier to reversion is larger for the adduct formed with N-methylmaleimide. The barrier to reversion for this adduct is relatively insensitive to changes in solvent while the adduct formed with maleic anhydride responds more strongly to changes in solvent polarity. The differences in reaction barrier and solvent dependence arises because the adduct formed with N-methylmalemide is more stable—leading to a larger barrier to reversion—while the adduct formed with maleic anhydride experiences a larger change in dipole during the reaction—leading to a larger solvent dependence.

## Introduction

Since the first report in 1928, the Diels-Alder cycloaddition has proven valuable as a model for understanding concerted reaction mechanisms and as a click reaction^1–3^. The Diels-Alder reaction is a [4 + 2] cycloaddition between a diene and dienophile which proceeds through a pericyclic transition state to form an adduct. In the context of its use in click chemistry, Diels-Alder chemistry is valued as a means to join together two chemicals in a fast and reliable manner. However, the utility of Diels-Alder chemistry is extended by its susceptibility to clean cycloreversion under relatively mild conditions. The accessibility of the retro Diels-Alder (rDA) reaction makes this class of reaction attractive for use in development of materials that require clean, reversible chemistry, such as for thermally remendable polymers^4–9^ and sensors^10^. A strength of the reaction for such applications is the fact that (as explained by the Woodward-Hoffman rules)^11^ the reaction is only activated thermally, and so is insensitive to light. Thus, remendable polymers built on this chemistry can be used outdoors without the need to worry about light-induced reversion.

Despite the reliance on the rDA for the above applications, the major focus of fundamental studies on Diels-Alder chemistry has historically been on the forward reaction. Though several reviews on the rDA reaction exist^12,13^, work on rDA reactions continues to lag behind that on the adduct forming direction. In order to enable the greatest control over desired materials properties, it is useful to understand how to control the cyclo-reversion. Learning to control the thermodynamics of the reversion dictates the conditions under which one can observe these behaviors, while learning to control the kinetics provides flexibility over how quickly the system responds.

**Figure 1:**
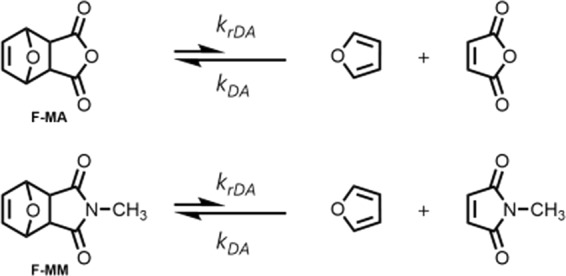
The reactions of interest. We studied the retro Diels-Alder reactions involving the adducts formed between furan and maleic anhydride (top) and furan and N-methylmaleimide (bottom). Focus was on the rate constant for the retro-reaction (${k}_{rDA}$). Drawings were made by the authors using ChemDraw.

This report seeks to add to the understanding of the control over the kinetics of the rDA reaction, exploring both structural and solvent effects. Using the common Diels-Alder diene, furan, we alter the structure of the dienophile through modification of a heteroatom: maleic anhydride versus *N*-methylmaleimide (Fig. 1). The resulting adducts of furan-maleic anhydride (F-MA) and furan-*N*-methylmaleimide (F-MM) are expected to have different barriers to rDA. Prior work on these systems suggests that the F-MM adduct is more stable than the F-MA adduct, and so has a larger barrier to cyclo-reversion^14^. This prior work was performed at a single temperature, and so the results were only given in terms of free energy ($\Delta G$) of the adduct, reactants, and transition state; and did not report on parameters that require temperature dependent experiments to determine—such as Arrhenius activation energy (${E}_{A}$), the enthalpy of activation (${\Delta }^{\ddagger }{H}^{\circ }$), and the entropy of activation (${\Delta }^{\ddagger }{S}^{\circ }$). All of these parameters should be sensitive to changes in the structure of the dienophile and solvent. Understanding how they change with structure and environment will increase the precision by which this reaction is employed in funcational materials.

We test the hypothesis that ${E}_{A}$, ${\Delta }^{\ddagger }{H}^{\circ }$, ${\Delta }^{\ddagger }{S}^{\circ }$ are sensitive to structural changes by determining the rate constant for reversion (${k}_{rDA}$, Fig. 1) at several temperatures and then using this data to extract values for ${E}_{A}$, ${\Delta }^{\ddagger }{H}^{\circ }$, and ${\Delta }^{\ddagger }{S}^{\circ }$. We find that both the Arrhenius activation energy and the enthalpy of activation respond to changes in structure. In addition, we reasoned that the structural differences between the dienophiles would result in differences in the polarity of the adduct and the transition state, which we would expect to lead to differences in solvent dependence of these activation parameters. We test this hypothesis by determining the activation parameters for these systems in different solvents and find that changes in solvent produces strong effects on these parameters for the F-MA adduct, but not the F-MM adduct.

## Results

### Choice of solvent

Prior to discussion of our results, it is worth remarking on the solvents we employed. Because we wished to follow the course of reaction using NMR, we were limited to solvents that we could obtain in deuterated form at reasonable cost. Because we wished to extract good estimates of ${E}_{A}$, ${\Delta }^{\ddagger }{H}^{\circ }$, and ${\Delta }^{\ddagger }{S}^{\circ }$, we also wished to examine the rate of reaction over a wide range of temperatures for which the reaction proceed at a reasonable rate, and this further restricted our solvents to those with higher boiling points. Of these, we obviously required solvents in which our adducts and their parent species were soluble. This limited our study to 1,1,2,2-tetrachloroethane (TCE), dimethyl sulfoxide (DMSO), and acetonitrile (ACN). The barrier to reaction for F-MM was large enough that the relatively low boiling point of ACN did not allow a sufficient temperature window for our experiments for this adduct. We also found that DMSO was not an innocent solvent for the F-MA reaction, and so we could not obtain meaningful kinetics for the F-MA reaction in DMSO. In the end, we were able to obtain reaction kinetics for rDA reactions of both adducts in TCE and for F-MA in ACN and F-MM in DMSO. Though this is a small set of solvents, they did allow us to test the main hypotheses that motivated this work: that structural and solvent changes would produce changes to the activation parameters of the rDA reaction, as shown and discussed below.

### Spectra

**Figure 2:**
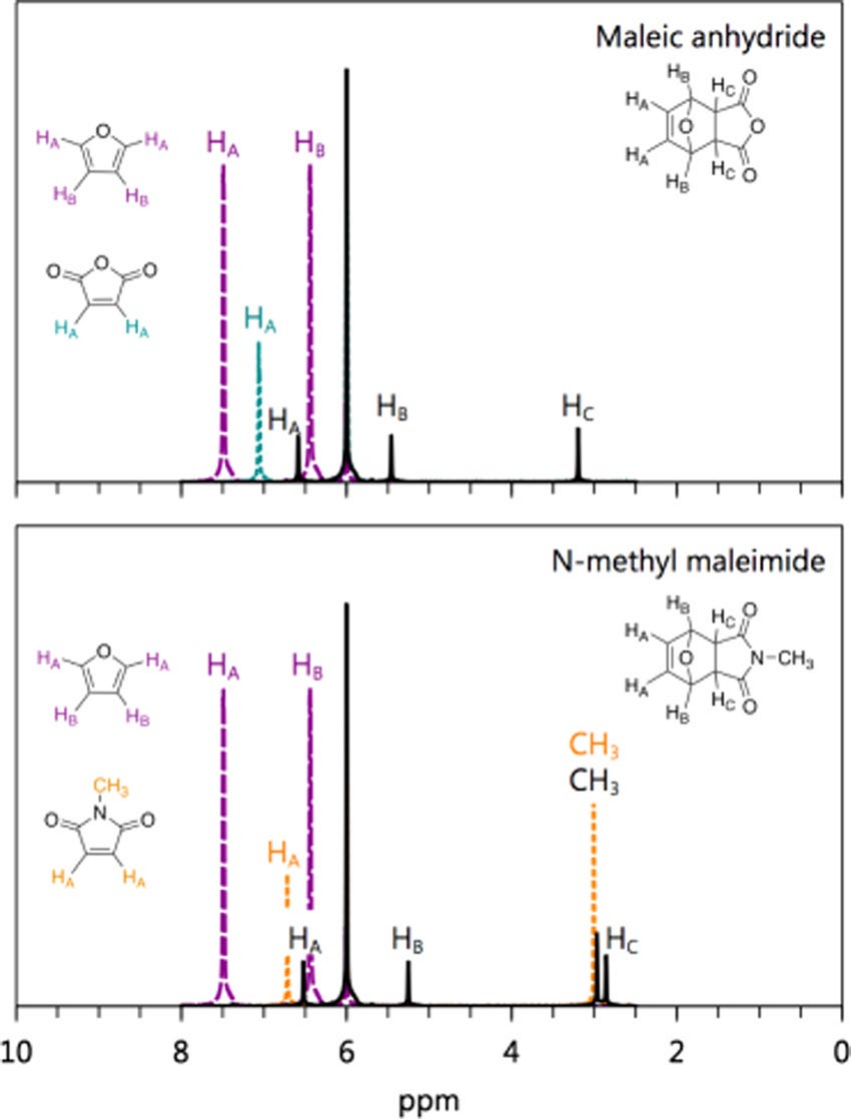
${}^{1}$H NMR spectra of the adducts (solid lines, black) and their constituent diene (dotted lines, purple) and dienophiles (dashed lines, teal and orange) in TCE. The top panel is for the species involving maleic anhydride (teal), while the bottom panel is for the species involving N-methyl maleimide (orange). In all spectra, the resonance at 6 ppm is associated with the solvent. The peaks are labeled by their associated protons on each structure.

We employed ${}^{1}$H NMR using a 300 MHz Bruker instrument to follow the course of the reaction. Figure 2 shows the NMR spectra of both adducts, as well as the furan and dienophile in TCE. As can be seen in this figure, the resonances associated with each species are well-resolved. This remains the case in all solvents used in our study, as can be seen in Fig. S1. Using these resonances, we followed the course of the rDA reaction and extracted rate constants for the reactions.

### Extraction of rate constant

**Figure 3:**
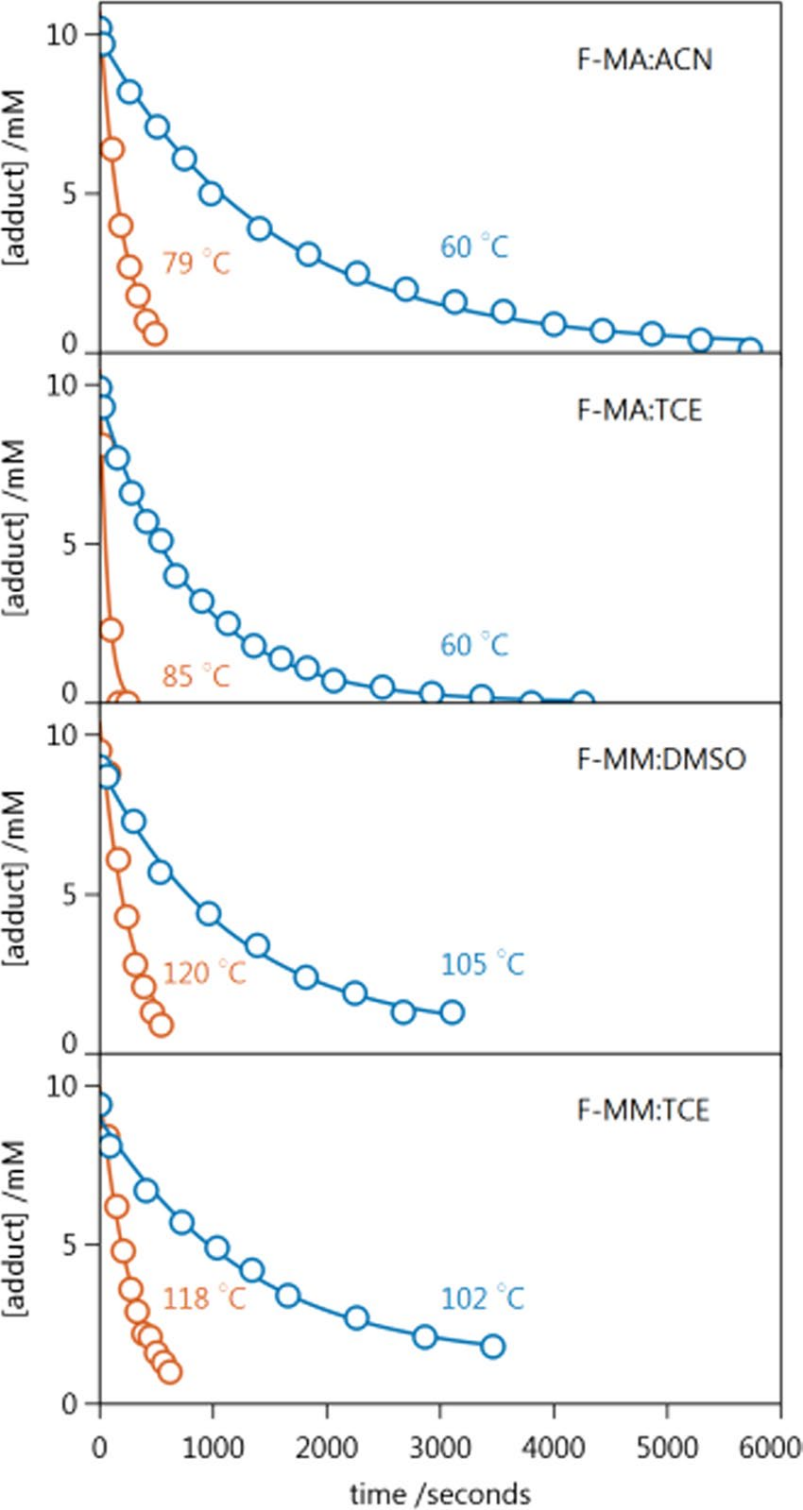
Kinetic traces for all four adduct:solvent systems. Shown for each are the data at the lowest (blue) and highest (orange) temperatures employed for that system. Also shown (solid lines) are the fits of these data to the kinetic model shown in Fig. 1.

To follow the course of the rDA reaction, we dissolved the adduct in the appropriate solvent at a concentration of ca. 10 mM, transferred this solution to an NMR tube, and then acquired a series of spectra over a window of between 10 and 100 minutes, depending on the rate of reaction for that solvent and temperature. During the course of the reaction, concentrations of the adduct, diene, and dienophile were obtained from the integrated ${}^{1}$H NMR peak areas. For this work, the focus is on the kinetic trace associated with the adduct, which decreases over time. For each adduct:solvent system we obtained such kinetic traces at at least six different temperatures. Figure 3 shows the concentration of the adduct as a function of time, obtained at the lowest and highest temperatures for each adduct:solvent system. The concentrations of the adduct obtained for all conditions are given in the Supplementary Information, Tables S1–S30.

In addition to the evolving concentration of the adduct, Fig. 3 shows fits of the data to the reaction mechanism shown in Fig. 1. These fits were obtained using Dynafit 4^15^, which returned values of both ${k}_{rDA}$ and ${k}_{DA}$ for each data set. For the lowest temperature conditions, both of these rate constants have reasonable values, indicating that the reaction proceeds to an equilibrium that is distinguishable from 100% reversion. However, at high temperatures, the reaction proceeds so strongly in the rDA direction that the values obtained for ${k}_{DA}$ are not meaningful. For this reason, we consider only the values of ${k}_{rDA}$ hereafter. The values of ${k}_{rDA}$ obtained from each kinetic trace are given in the Supplementary Data, Tables S31–34.

### Extraction of kinetic parameters

**Figure 4:**
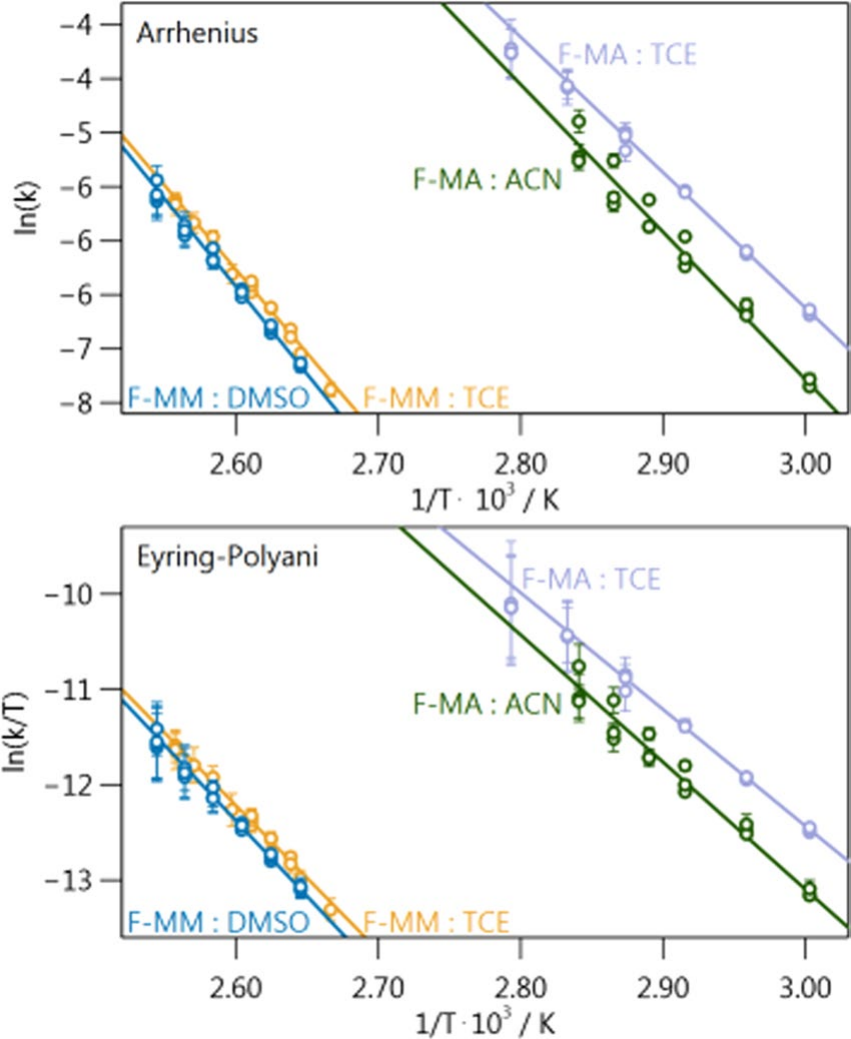
Arrhenius and Eyring-Polanyi plots of our rate constants extracted from the kinetic traces. The error bars associated with each point are the standard errors of the rate constants, obtained by the fits shown in Fig. 3.

The rate constants we obtained at different temperatures can be used to calculate kinetic parameters, such as activation energy (${E}_{A}$), frequency factor (${\nu }_{N}$), enthalpy of activation (${\Delta }^{\ddagger }{H}^{\circ }$), and entropy of activation (${\Delta }^{\ddagger }{S}^{\circ }$), using both Arrhenius (ln$({k}_{rDA})$ vs. ${T}^{-1}$) and Eyring-Polanyi (ln(${k}_{rDA}/T$) vs. ${T}^{-1}$) plots. For both figures, the data is fit to a straight line. The Arrhenius plot (Fig. 4, upper panel) yields ${E}_{A}$ and ${\nu }_{N}$ from the slope and intercept, respectively, as shown in Equations 1 and 2,

1 $${E}_{A}=-\,R\cdot slope$$

2 $${\nu }_{N}=exp(intercept)$$

where R is the ideal gas constant. The Eyring-Polanyi plot (Fig. 4, lower panel) yields ${\Delta }^{\ddagger }{H}^{\circ }$ and ${\Delta }^{\ddagger }{S}^{\circ }$ from the slope and intercept respectively, as shown in Equations 3 and 4,

3 $${\Delta }^{\ddagger }{H}^{\circ }=-\,R\cdot slope$$

4 $${\Delta }^{\ddagger }{S}^{\circ }=R\left(intercept-ln\frac{{k}_{b}}{h}\right)$$

where ${k}_{b}$ and $h$ are the Boltzmann constant and Planck’s constant, respectively. Figure 4 shows linear regressions fit to our data for each adduct:solvent pair. The focus of this work is on the activation barrier, enthalpy of activation, and entropy of activation. The values of these parameters that we obtained from these fits are given in Table 1. The standard errors in these parameters are obtained from the regression. The frequency factor is not discussed herein, but its values can be found in Table S35.

**Table 1:** Values, and associated standard errors, for ${E}_{A}$, ${\Delta }^{\ddagger }{S}^{\circ }$, and ${\Delta }^{\ddagger }{S}^{\circ }$ for all four adduct:solvent systems. These values (and their standard errors) were obtained from the linear regressions shown in Fig. 4.

Adduct:Solvent	${{\boldsymbol{E}}}_{{\boldsymbol{A}}}$ /kJ $\cdot$ mol${}^{-1}$	${\boldsymbol{\Delta }}{{\boldsymbol{H}}}^{{\boldsymbol{\dagger }}}$ /kJ $\cdot$ mol${}^{-1}$	${\boldsymbol{\Delta }}{{\boldsymbol{S}}}^{{\boldsymbol{\dagger }}}$ /J $\cdot$ mol${}^{-1}$
F-MA:TCE	$104\pm 1$	$102\pm 3$	$178\pm 7$
F-MA:ACN	$114\pm 1$	$111\pm 2$	$200\pm 6$
F-MM:TCE	$129\pm 4$	$126\pm 8$	$203\pm 18$
F-MM:DMSO	$134\pm 4$	$132\pm 7$	$215\pm 17$

## Discussion

The chemical systems we employed were selected to examine how activation parameters for rDA reactions (${E}_{A}$, ${\Delta }^{\ddagger }{H}^{\circ }$, ${\Delta }^{\ddagger }{S}^{\circ }$) would respond to two different types of changes: structural changes within the dienophile and changes in the polarity of the solvent. Using the F-MA:TCE and F-MM:TCE systems, we isolated the effects of alteration of the dienophile while keeping the diene (furan) and solvent constant. Due to the high temperatures employed, the selection of the solvents was limited to those which simultaneously satisfied the requirements for adduct solubility, availability in deuterated form, and those possessing a high boiling point. The resulting set of solvents were small, but did enable us to comment on the effect of changes in polarity. Both of these alterations—structure and polarity of environment—constitute design parameters for the practical use of rDA reactions, and so are parameters for which it is useful to understand their influence over the activation parameters of the reaction.

All four of the adduct:solvent systems we examined show significant barriers to rDA reactions, with ${E}_{A}$ barriers always larger than 100 kJ/mol, even when taking into consideration the standard error associated with the estimates of this parameter. The majority of the barrier comes from enthalpic effects (${\Delta }^{\ddagger }{H}^{\circ }$). We also find ${\Delta }^{\ddagger }{S}^{\circ }$ is positive for each transition state, which is consistent with the reaction being dissociative. All three activation parameters respond to changes in structure of the adduct and polarity of solvent, and we consider both of these dependencies in turn.

### Effects of structural changes

Acquiring values for the activation parameters of both adducts in TCE isolates the effects of structural changes to the dienophile. Examination of the parameters in this solvent clearly show that the barrier to rDA reaction increases significantly when the dienophile is changed from maleic anhydride to N-methylmaleimide. This increase is seen for both ${E}_{A}$ and ${\Delta }^{\ddagger }{H}^{\circ }$, while ${\Delta }^{\ddagger }{S}^{\circ }$ does not experience a change outside of the standard errors for this parameter, showing that the primary effect of the structural change is enthalpic in nature. The natural question to ask is if this barrier increase is due to a destabilization of the transition state, a stabilization of the DA adduct, or both. Prior work by DiMare and Rickborn comparing the thermodynamics of F-MA and F-MM systems in ACN concluded that the transition state lay a similar height above the separate diene and dienophile^14^. That is, the transition state for the forward DA is similar for both complexes. However, this prior work also reported that the F-MM adduct was more thermodynamically stable than the F-MA adduct by $\sim$12 kJ/mol—which would account for roughly half the difference in transition state energy we observe in TCE. The other half can be explained by solvation (*vide infra*).

This prior work also provided an explanation for the extra stability of the F-MM adduct over the F-MA adduct. Namely, using the heats of hydrogenation of the double bond in the dienophiles as a proxy for the the energy of C-C bond formation, they found that the calculated heats of hydrogenation for maleic anhydride is $\sim$10.1 kJ/mol less than that of N-methylmaleimide, and concluded that this was the primary source of the added stability of the F-MM adduct. The fact that we observe only ${E}_{A}$ and ${\Delta }^{\ddagger }{H}^{\circ }$ to undergo substantial change upon changes to the dienophile provides justification for this enthalpic explanation.

The extra height of the reaction barrier for F-MM can also be understood in terms of standard rules for Diels-Alder chemistry, which state that reactivity is improved by adding electron withdrawing groups to the dienophile. The anhydride moiety of maleic anhydride is a better withdrawing group than the N-methyl imide of the N-methylmaleimide. Thus, one would expect a larger barrier for reactions involving the N-methylmaleimide than for the maleic anhydride. Though the reasoning of DiMare and Rickborn is perhaps more compelling, it is satisfying that our observations are consistent with this rule-of-thumb for Diels-Alder reactions.

### Effects of changes in solvent

Examination of Table 1 shows that the activation parameters for the F-MA adduct responds more strongly to changes in solvent than do those for the F-MM adduct. The Hughes-Ingold rule^16^ stipulates that we can expect increases in the barrier to reaction upon increasing solvent polarity when the transition state is less polar than the reactants—a statement that is also consistent with standard solvation theories, such as the Onsager model^17^. For our solvents, TCE is the least polar, and DMSO with most, with the dipole moment of the solvent molecules being 1.33 D for TCE, 3.44 D for ACN, and 4.1 D for DMSO. Thus, to understand the response of the activation parameters of F-MA to changes in polarity, and the relative insensitivity of those of F-MM, it would be useful to consider the polarity of these two adducts, as well as their transition states.

**Figure 5:**
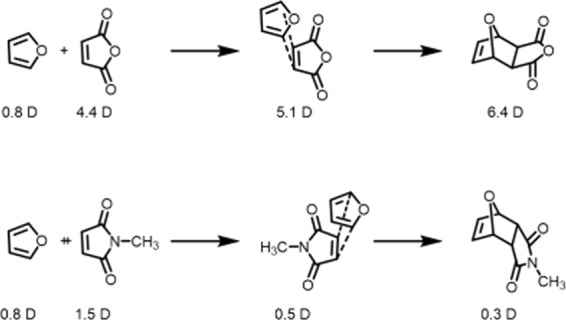
Estimated dipoles for the various species involved in the Diels-Alder reaction. These values are taken from MP2 quantum chemical calculations, as detailed in the experimental section. Drawings were made by the authors using ChemDraw.

Because both reactions involve furan, differences in polarity for the adducts and their associated transition states will arise primarily from differences in the dienophile. While we could not find experimental values for the dipoles of these species, an MP2 quantum mechanical calculation (performed in Orca) provides estimated values of 4.4 D and 1.5 D for maleic anhydride and N-methylmaleimide, respectively (Fig. 5). Using the same methods, we calculated the dipole moment for the adducts. In doing so, it was important to account for the different conformers formed by F-MA and F-MM. The Diels-Alder reaction that forms these adducts can yield either *endo* or *exo* products—which vary based upon the relative orientation of the diene and dienophile in the transition state. The spectra shown in Fig. 2 have only a single set of resonances for the adducts, indicating that for F-MA and F-MM, only one of the possible conformers is present in measurable quantities. In general, DA adducts form in *endo* geometry preferentially, however prior work shows that the F-MA predominately forms the *exo* conformer, while F-MM forms the *endo* conformer (Fig. 5). In general, for these two dienophiles and furan, it is the *exo* conformer that is the more polar of the two. As a result, the difference between the polarities of the adducts is even greater than for their parent dienophiles, with F-MA and F-MM having calculated dipoles of 6.4 D and 0.3 D, respectively (Fig. 5). Finally, though an experimentally observed value of the dipole for furan is known (0.67 D)^18^, we also calculated the dipole of furan, in order to test the validity of our approach. Our calculations provide a dipole moment of 0.8 D for the gas phase species, which seems in reasonable enough agreement to permit the qualitative discussion below.

For the transition state, the expected geometry for the *exo* forming F-MA is such that the dipole of the furan will align with that of the dienophile, while for the *endo* forming F-MM the dipoles of the dienophile and furan will oppose one another. Using our calculated value for the dipole of furan and the dienophiles, we find that the extreme limits for the additive dipoles of the transition state would be 5.1 D and 0.5 D for F-MA and F-MM, respectively (Fig. 5). Thus, we find a greater than four-fold difference in the estimated change in dipole size between adduct and transition state for F-MA (${D}_{adduct}-{D}_{transitionstate}=1.3D$) than F-MM (${D}_{adduct}-{D}_{transitionstate}=0.3D$). It should be noted that the difference between these numbers and those which would be arrived at using the values reported in Figure 5 owe to the effects of rounding and significant figures.  We want to emphasize that the methods that we use are by no means meant to be quantitatively accurate; however, we do feel that they are sufficient to state that, when comparing F-MA to F-MM, the F-MA adduct and transition state are both more polar and that this adduct experiences a larger change in polarity during the rDA reaction.

The calculated larger change in polarity during the rDA reaction supports the observation that the activation parameters of F-MA respond more strongly to changes in solvent polarity. This result is also compounded by the fact that both the F-MA adduct and its transition state are more polar than for F-MM. The Onsager model of solvation predicts that the energy of solvation is related to the *square* of the solute’s dipole moment^17^, and so the more polar species associated with the F-MA adduct should respond more strongly to changes in solvent than the F-MM adduct—which is exactly what we find. Though both species do experience increases in the barrier to activation upon moving from TCE to a more polar solvent, the changes to the barrier for F-MM are within the standard error associated with the estimation of each parameter found in Table 1, while all the changes to the barrier for F-MA that accompany changes in solvent lie outside the standard error associated with the parameter estimate. This reasoning is further supported by the fact that we find that the change in activation parameters is greatest for the F-MA system, despite the fact that the difference in polarity between TCE and ACN (2.11 D) is 24% less than that between TCE and DMSO (2.77 D). Finally, we note that the direction of the shift in activation parameters (larger barriers in more polar solvents) are consistent with the larger calculated dipole moments for both adducts compared to their transition states.

When changing solvents, we also find the only significant change in ${\Delta }^{\ddagger }{S}^{\circ }$ for our experiments, occurs for the F-MA adduct. As our calculations indicate that both the adduct and transition state of F-MA are more polar than for F-MM, this finding is reasonable. With larger polarity of the solute, we expect there to be better ordering of the solvent molecules around the solute. Thus, when changing the polarity of the solvent, we also expect to see the largest change in ordering *between* solvents—supporting a large change in ${\Delta }^{\ddagger }{S}^{\circ }$ when moving from a less polar (TCE) solvent to a more polar (ACN) solvent. In addition, we would expect a larger ${\Delta }^{\ddagger }{S}^{\circ }$ for systems with larger dipole moments, which is precisely what we find.

Finally, given the above discussion of solvation energy, we can return to consideration of the prior reports of barriers for rDA of F-MA and F-MM in ACN^14^. This prior report gave the free energy barrier to rDA for F-MA as 109 kJ/mol in ACN at $4{0}^{\circ }$C. Examination of Table 1 shows that our estimated value of the ${E}_{A}$ and ${\Delta }^{\ddagger }{H}^{\circ }$ in ACN is similar to this prior reported value, and suggests that the disparity between the barrier to reaction in TCE versus ACN noted above is likely due to changes in solvation energy upon moving from the more polar ACN to the less polar TCE.

## Conclusions

Using temperature dependent NMR, we have followed the rate of two retro Diels-Alder reactions in three different solvents. The retro Diels-Alder reactions involved two different adducts, formed between furan and either maleic anhydride or N-methylmaleimide. This work allowed us to determine the activation parameters for the reactions. We find that the activation parameters are dependent on the structure of the Diels-Alder adducts, with the barrier to reversion being larger for the N-methylmaleimide, due to the fact that the adduct is more stable than the adduct formed with maleic anhydride. However, the adduct formed with maleic anhydride is more polar, and experiences a larger change in polarity upon attaining the transition state. For this reason, the maleic anhydride displays a larger dependence on solvent than does the N-methylmaleimide adduct. In total, this work shows clear dependencies of the retro Diels-Alder reaction on both structural and environmental effects and provides insight that can help in the selection of such reactions for use in functional materials.

## Experimental

### Materials

The reagents were obtained from the following suppliers, and used as received. Furan (Alfa Aesar), maleic anhydride (Fluka), N-methylmaleimide (TCI), 1,1,2,2-tetrachloroethane-d2 (Acros), acetonitrile-d3 (Alfa Aesar), and dimethyl sulfoxide-d6 (Magnisolv). Reagents were used as recieved, without further purification.

### Synthesis of adduct

The F-MA adduct was synthesized as in a prior report by us^19^. A solution of 3 M furan and 3 M maleic anhydride in dichloromethane was left to stand under ambient laboratory conditions overnight. The crystals were collected using vacuum filtration, and washed with cold dichloromethane. The formation of the adduct was confirmed using 1H NMR. The F-MM adduct was synthesized following the procedure from Anderson and Milowsky^20^.

### Spectroscopic details

All NMR were collected on the same instrument, a Bruker CDPX-300. Integrated intensities of the resonances were obtained by fitting in MestReNova.

### Fitting of kinetic traces

Kinetics were extracted using DynaFit 4^15^. This program using numerical methods to obtain best fits of kinetic models to data. The program returns both parameters of best fit, as well as the standard errors associated with these estimates.

### Calculation of dipole moments

The dipole moments of the dienophiles and adducts were estimated in ORCA 4.1 using Møller–Plesset perturbation theory (MP2). The starting structure for the calculation was a structure optimized in Avogadro v 1.2.0 using a MMFF94 force field using a steepest decent algorithm.

## Supplementary information

Supplementary Information

## Data Availability

All data analysed during this study are included in this published article (and its Supplementary Information files). Any data of interest not found in these files (e.g., raw IR spectra, computational output files, etc.) are available from the corresponding author on reasonable request.
