# Supplementary material for: Structural and solvent control over activation parameters for a pair of retro Diels-Alder reactions
Source: Sci Rep. 2019 Dec 4;9:18267. doi: 10.1038/s41598-019-54156-4 (PMC6892874; doi:10.1038/s41598-019-54156-4)
Supplement: Supplementary file 1 — Supplementary Information [file 41598_2019_54156_MOESM1_ESM.pdf]

Structural and solvent control over activation parameters for a pair of retro Diels-Alder reactions

Andrea L. Widstrom and Benjamin J. Lear

Supplementary Information:

1. Synthetic methods
2. Experimental methods
3. Integrated peak areas and concentrations
4. Calculated rate constants
5. Extracted kinetic parameters
6. References

1. Synthetic methods

The reagents and solvents were all obtained commercially, and used as received. All three adducts were made with furan as the diene. The maleic anhydride adduct was synthesized by combining a 3 M solution of maleic anhydride in dichloromethane with a 3 M solution of furan in dichloromethane, and allowed to sit overnight. The crystals were then vacuum filtered and washed with cold dichloromethane. The maleimide adduct was synthesized using the same procedure. The n-methyl maleimide adduct was synthesized following the procedure from Anderson and Milowsky[1].

2. Experimental methods

Solutions of 0.008 M adduct in the solvent of choice were prepared, and 0.5 ml of the stock was placed in an NMR tube. Spectra were collected with ^{1}H NMR on a Bruker CDPX-300 running WinXNMR, using a variable temperature probe. Temperatures were selected based on the measured reversion temperature of the adducts. Each set of solutions were run back to back on the same day to minimize variations, with all sets measured in triplicate, except the n-methyl maleimide adduct which was measured five times due to instrumental difficulties. A sample experiment is shown in Figure S1, for the furan-maleic anhydride adduct in tetrachloroethane (TCE).

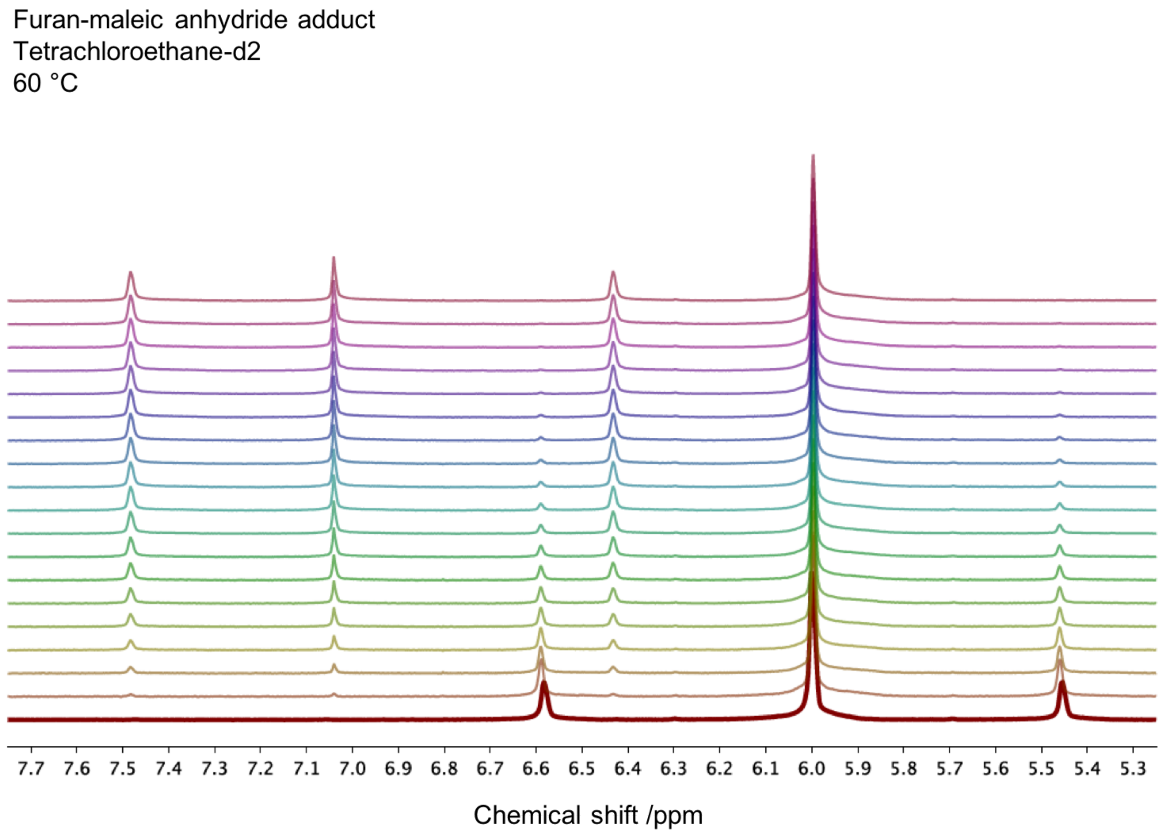

Figure S1: ^{1}H NMR of furan-maleic anhydride adduct in TCE undergoing reversion at 60 C. Solvent peak at 6 ppm. Adduct peaks shown at 6.56 and 5.46 ppm. Furan peaks shown at 7.43 ppm, and maleic anhydride at 7.03 and 6.46 ppm.

3. Integrated peak areas and calculated concentrations:

Peak areas were normalized to the furan 7.43 ppm peak. The ratio is the average of the adduct peak areas after normalization. All peaks were 1:1. At time 0, the ratio is listed as inf as a placeholder as there was no furan present in the solution, and the peak ratio was undefined.

Table S1. Ratio of NMR integrated peak areas and concentration of the adduct as a function of time at 60°C for the furan-maleic anhydride adduct in TCE. Three trials are present.

60°C								
Time (sec)	Ratio	FMA (M)	Time (sec)	Ratio	FMA (M)	Time (sec)	Ratio	FMA (M)
0	inf	0.0099	0	inf	0.0088	0	inf	0.0087
30	16.23	0.0093	28	18.27	0.0083	33	22.99	0.0083
156	3.49	0.0077	155	3.99	0.0070	167	4.28	0.0070
281	2.06	0.0066	280	2.22	0.0061	292	2.16	0.0059
414	4.16	0.0057	408	1.4233	0.0052	419	1.4	0.0051
540	1.06	0.0051	534	1.02	0.0044	544	1.01	0.0044
672	0.67	0.0040	660	0.793	0.0039	671	0.747	0.0037
900	0.483	0.0032	795	0.547	0.0031	802	0.53	0.0030
1130	0.333	0.0025	1023	0.3833	0.0024	1028	0.35	0.0022
1358	0.223	0.0018	1256	0.278	0.0019	1257	0.247	0.0017
1598	0.16	0.0014	1484	0.18	0.0013	1485	0.15	0.0011
1827	0.12	0.0011	1716	0.143	0.0011	1716	0.12	0.0009
2062	0.077	0.0007	1945	0.107	0.0008	1954	0.087	0.0007
2491	0.05	0.0005	2178	0.0767	0.0006	2190	0.0567	0.0005
2926	0.033	0.0003	2604	0.05	0.0004	2620	0.03	0.0003
3364	0.02	0.0002	3032	0.037	0.0003	3050	0.0167	0.0001
3803	0	0.0000	3461	0.02	0.0002	3480	0.0166	0.0001
4254	0	0.0000	3893	0.01	0.0001	3911	0.01	0.0001

Table S2. Ratio of NMR integrated peak areas and concentration of the adduct as a function of time at 65°C for the furan-maleic anhydride adduct in TCE. Three trials are present.

65°C								
Time (sec)	Ratio	FMA (M)	Time (sec)	Ratio	FMA (M)	Time (sec)	Ratio	FMA (M)
0	inf	0.0099	0	inf	0.0088	0	inf	0.0087
28	9.03	0.0089	30	8.95	0.0079	26	12.1	0.0080
152	2.06	0.0066	160	2.19	0.0060	180	2.16	0.0059
279	1.09	0.0051	287	1.08	0.0046	285	1.06	0.0045
407	0.653	0.0039	412	0.657	0.0035	411	0.647	0.0034
534	0.427	0.0030	536	0.447	0.0027	536	0.43	0.0026
658	0.283	0.0022	662	0.31	0.0021	662	0.293	0.0020
784	0.2	0.0016	789	0.23	0.0016	807	0.18	0.0013
910	0.143	0.0012	914	0.147	0.0011	934	0.13	0.0010
1036	0.11	0.0010	1040	0.107	0.0008	1059	0.0967	0.0008
1173	0.0933	0.0008	1164	0.09	0.0007	1186	0.083	0.0007
1300	0.07	0.0006	1291	0.063	0.0005	1312	0.0567	0.0005
1426	0.06	0.0006	1415	0.0533	0.0004	1439	0.0467	0.0004
1554	0.0467	0.0004	1540	0.0467	0.0004	1564	0.04	0.0003
1680	0	0.0000	1665	0.03	0.0003	1690	0.01	0.0001
1811	0	0.0000	1800	0.01	0.0001	1830	0	0.0000
1948	0	0.0000	1930	0	0.0000	1955.5	0	0.0000

Table S3. Ratio of NMR integrated peak areas and concentration of the adduct as a function of time at 70°C for the furan-maleic anhydride adduct in TCE. Three trials are present.

70°C								
Time (sec)	Ratio	FMA (M)	Time (sec)	Ratio	FMA (M)	Time (sec)	Ratio	FMA (M)
0	inf	0.0099	0	inf	0.0088	0	inf	0.0087
30	12.15	0.0091	25	10.72	0.0080	29	12.08	0.0080
101	2.73	0.0072	98	2.72	0.0064	102	2.25	0.0060
175	1.08	0.0051	173	1.12	0.0046	178	1.14	0.0046
250	0.62	0.0038	248	0.63	0.0034	252	0.62	0.0033
325	0.42	0.0029	321	0.393	0.0025	326	0.4	0.0025
423	0.223	0.0018	396	0.263	0.0018	401	0.267	0.0018
498	0.163	0.0014	469	0.18	0.0013	475	0.18	0.0013
573	0.12	0.0011	544	0.123	0.0010	549	0.14	0.0011
656	0.0767	0.0007	620	0.0867	0.0007	625	0.107	0.0008
733	0.0667	0.0006	695	0.067	0.0006	701	0.073	0.0006
808	0.033	0.0003	770	0.0433	0.0004	777	0.053	0.0004
884	0	0.0000	848	0.0367	0.0003	856	0.037	0.0003
965	0	0.0000	932	0	0.0000	932	0	0

Table S4. Ratio of NMR integrated peak areas and concentration of the adduct as a function of time at 75°C for the furan-maleic anhydride adduct in TCE. Three trials are present.

75°C								
Time (sec)	Ratio	FMA (M)	Time (sec)	Ratio	FMA (M)	Time (sec)	Ratio	FMA (M)
0	inf	0.0099	0	inf	0.0088	0	inf	0.0087
28	11.97	0.0091	28	8.97	0.0079	25	8.38	0.0077
102	1.72	0.0062	101	1.39	0.0051	99	1.41	0.0051
175	0.683	0.0040	175	0.463	0.0028	173	0.493	0.0029
250	0.36	0.0026	249	0.213	0.0015	250	0.22	0.0016
323	0.163	0.0014	324	0.107	0.0008	322	0.113	0.0009
396	0.09	0.0008	396	0.0567	0.0005	397	0.07	0.0006
472	0	0.0000	471	0	0.0000	474	0.017	0.0001
548	0	0.0000	547	0	0.0000	549	0	0

Table S5. Ratio of NMR integrated peak areas and concentration of the adduct as a function of time at 80°C for the furan-maleic anhydride adduct in TCE. Three trials are present.

80°C								
Time (sec)	Ratio	FMA (M)	Time (sec)	Ratio	FMA (M)	Time (sec)	Ratio	FMA (M)
0	inf	0.0099	0	inf	0.0088	0	inf	0.0087
28	6.55	0.0086	30	4.74	0.0073	25	5.49	0.0073
101	0.63	0.0038	104	0.6267	0.0034	99	0.68	0.0035
175	0.17	0.0014	178	0.15	0.0011	173	0.16	0.0012
250	0.0467	0.0004	251	0.05	0.0004	248	0.05	0.0004
322	0	0.0000	327	0	0	322	0	0

Table S6. Ratio of NMR integrated peak areas and concentration of the adduct as a function of time at 85°C for the furan-maleic anhydride adduct in TCE. Three trials are present.

85C								
Time (sec)	Ratio	FMA (M)	Time (sec)	Ratio	FMA (M)	Time (sec)	Ratio	FMA (M)
0	inf	0.0099	0	inf	0.0088	0	inf	0.0087
25	4.51	0.0081	33	3.03	0.0066	29	3.36	0.0067
100	0.303	0.0023	107	0.2367	0.0017	102	0.293	0.0020
172	0	0.0000	181	0.03	0.0003	177	0.02	0.0002
246	0	0.0000	255	0	0.0000	252	0	0

Furan-maleic anhydride, acetonitrile:

Table S7. Ratio of NMR integrated peak areas and concentration of the adduct as a function of time at 60°C for the furan-maleic anhydride adduct in acetonitrile. Three trials are present.

60°C								
Time (sec)	Ratio	FMA (M)	Time (sec)	Ratio	FMA (M)	Time (sec)	Ratio	FMA (M)
0	inf	0.0114	0	inf	0.0102	0	inf	0.0119
30	51.76	0.0112	28	18.987	0.0097	31	7.28	0.0105
166	10.03	0.0104	263	4.03	0.0082	467	1.83	0.0077
293	4.67	0.0094	507	2.26	0.0071	896	0.99	0.0059
421	3.15	0.0087	746	1.48	0.0061	1324	0.637	0.0046
546	2.48	0.0082	980	0.97	0.0050	1752	0.427	0.0036
671	1.94	0.0075	1408	0.627	0.0039	2180	0.303	0.0028
804	1.41	0.0067	1839	0.44	0.0031	2610	0.223	0.0022
1031	1.06	0.0059	2269	0.317	0.0025	3039	0.153	0.0016
1259	0.797	0.0051	2698	0.237	0.0020	3469	0.113	0.0012
1487	0.64	0.0045	3128	0.187	0.0016	3896	0.09	0.0010
1715	0.52	0.0039	3556	0.14	0.0013	4323	0.07	0.0008
1944	0.43	0.0034	4001	0.093	0.0009	4753	0.05	0.0006
2180	0.33	0.0028	4429	0.07	0.0007	-	-	-
2610	0.247	0.0023	4866	0.063	0.0006	-	-	-
3040	0.193	0.0019	5292	0.037	0.0004	-	-	-
3470	0.14	0.0014	5730	0.01	0.0001	-	-	-

Table S8. Ratio of NMR integrated peak areas and concentration of the adduct as a function of time at 65°C for the furan-maleic anhydride adduct in acetonitrile. Three trials are present.

65°C								
Time (sec)	Ratio	FMA (M)	Time (sec)	Ratio	FMA (M)	Time (sec)	Ratio	FMA (M)
0	inf	0.0114	0	inf	0.0102	0	inf	0.0119
30	18.36	0.0108	31	28.49	0.0099	27	21	0.0114
164	3.83	0.0091	168	3.6	0.0080	163	2.87	0.0088
292	2.01	0.0076	396	1.49	0.0061	392	1.9197	0.0078
418	1.31	0.0065	635	0.857	0.0047	617	0.72	0.0050
546	0.98	0.0057	868	0.487	0.0034	851	0.39	0.0033
679	0.69	0.0047	1299	0.267	0.0022	1283	0.187	0.0019
907	0.43	0.0034	1728	0.13	0.0012	1712	0.103	0.0011
1135	0.323	0.0028	2190	0.07	0.0007	2140	0.06	0.0007
1366	0.207	0.0020	2618	0.043	0.0004	2569	0.04	0.0005
1593	0.167	0.0016	3050	0.02	0.0002	2998	0.027	0.0003
1824	0.083	0.0009	3485	0.01	0.0001	3426	0	0.0000
2058	0.063	0.0007	-	-	-	3858	0	0.0000

Table S9. Ratio of NMR integrated peak areas and concentration of the adduct as a function of time at 70°C for the furan-maleic anhydride adduct in acetonitrile. Three trials are present.

70°C								
Time (sec)	Ratio	FMA (M)	Time (sec)	Ratio	FMA (M)	Time (sec)	Ratio	FMA (M)
0	inf	0.0114	0	inf	0.0102	0	inf	0.0119
134	4.01	0.0092	21	54.6	0.0100	34	23.29	0.0114
211	2.15	0.0078	105	5.79	0.0087	113	4.02	0.0095
289	1.34	0.0066	180	2.58	0.0074	187	1.94	0.0079
365	0.993	0.0057	255	1.553	0.0062	268	1	0.0060
441	0.72	0.0048	334	1.057	0.0053	395	0.567	0.0043
515	0.58	0.0042	412	0.723	0.0043	522	0.357	0.0031
593	0.457	0.0036	537	0.483	0.0033	648	0.243	0.0023
666	0.38	0.0032	666	0.347	0.0026	774	0.153	0.0016
749	0.303	0.0027	797	0.243	0.0020	970	0.097	0.0011
875	0.237	0.0022	929	0.163	0.0014	1139	0.053	0.0006
1004	0.15	0.0015	1162	0.07	0.0007	1365	0.03	0.0003
1130	0.13	0.0013	1391	0.047	0.0005	1591	0.02	0.0002
1259	0.073	0.0008	1619	0.023	0.0002	1818	0.001	0.0000
1386	0.057	0.0006	1846	0.001	0.0000	2046	0	0.0000

Table S10. Ratio of NMR integrated peak areas and concentration of the adduct as a function of time at 73°C for the furan-maleic anhydride adduct in acetonitrile. Three trials are present.

73°C								
Time (sec)	Ratio	FMA (M)	Time (sec)	Ratio	FMA (M)	Time (sec)	Ratio	FMA (M)
0	inf	0.0114	0	inf	0.0102	0	inf	0.0119
29	32.12	0.0111	27	32.9	0.0099	28	20.51	0.0114
106	3.86	0.0091	114	3.84	0.0081	109	2.67	0.0087
181	1.73	0.0072	189	1.66	0.0064	182	1.22	0.0066
257	1.047	0.0059	265	0.977	0.0051	258	0.697	0.0049
334	0.67	0.0046	339	0.657	0.0041	332	0.443	0.0037
411	0.5	0.0038	414	0.47	0.0033	405	0.307	0.0028
485	0.37	0.0031	490	0.343	0.0026	480	0.22	0.0021
565	0.267	0.0024	567	0.26	0.0021	554	0.147	0.0015
640	0.22	0.0021	641	0.193	0.0017	630	0.107	0.0012
719	0.127	0.0013	720	0.15	0.0013	706	0.077	0.0009
795	0.09	0.0009	796	0.123	0.0011	782	0.067	0.0007
870	0.06	0.0006	873	0.07	0.0007	857	0.047	0.0005
-	-	-	947	0.053	0.0005	935	0.01	0.0001
-	-	-	1025	0.037	0.0004	1005	0	0.0000

Table S11. Ratio of NMR integrated peak areas and concentration of the adduct as a function of time at 76°C for the furan-maleic anhydride adduct in acetonitrile. Three trials are present.

76°C								
Time (sec)	Ratio	FMA (M)	Time (sec)	Ratio	FMA (M)	Time (sec)	Ratio	FMA (M)
0	inf	0.0114	0	inf	0.0102	0	inf	0.0119
27	25.24	0.0110	26	27.62	0.0099	27	18.98	0.0113
103	3.12	0.0087	112	2.64	0.0074	107	1.907	0.0078
179	1.22	0.0063	187	1.12	0.0054	182	0.733	0.0050
254	0.73	0.0048	262	0.663	0.0041	256	0.36	0.0032
328	0.47	0.0037	337	0.427	0.0031	331	0.207	0.0020
404	0.38	0.0032	412	0.28	0.0022	405	0.123	0.0013
480	0.25	0.0023	489	0.207	0.0018	482	0.08	0.0009
555	0.177	0.0017	563	0.123	0.0011	555	0.047	0.0005
630	0.067	0.0007	636	0.083	0.0008	631	0.01	0.0001
-	-	-	714	0.053	0.0005	709	0	0.0000

Table S12. Ratio of NMR integrated peak areas and concentration of the adduct as a function of time at 60°C for the furan-maleic anhydride adduct in acetonitrile. Three trials are present.

79°C								
Time (sec)	Ratio	FMA (M)	Time (sec)	Ratio	FMA (M)	Time (sec)	Ratio	FMA (M)
0	inf	0.0114	0	inf	0.0102	0	inf	0.0119
28	16.69	0.0108	27	18.73	0.0097	28	12.67	0.0110
103	1.83	0.0074	111	1.65	0.0064	114	0.96	0.0058
179	0.71	0.0047	186	0.64	0.0040	190	0.31	0.0028
256	0.36	0.0030	263	0.35	0.0027	265	0.13	0.0014
330	0.23	0.0021	338	0.213	0.0018	339	0.06	0.0007
404	0.097	0.0010	413	0.113	0.0010	414	0.03	0.0003
481	0	0.0000	488	0.06	0.0006	490	0.01	0.0001

Furan- n-methyl maleimide adduct, TCE

Table S13. Ratio of NMR integrated peak areas and concentration of the adduct as a function of time at 102°C for the furan - N-methyl maleimide adduct in TCE. A single trials is present.

102°C		
Time (sec)	Ratio	FMA (M)
0	inf	0.0094
90	6.53	0.0081
410	2.56	0.0067
724	1.555	0.0057
1035	1.075	0.0049
1342	0.8	0.0042
1659	0.575	0.0034
2264	0.4	0.0027
2865	0.285	0.0021
3464	0.235	0.0018

Table S14. Ratio of NMR integrated peak areas and concentration of the adduct as a function of time at 105°C and 106°C for the furan - N-methyl maleimide adduct in TCE. A total of three trials are present.

105°C			106°C					
Time (sec)	Ratio	FMA (M)	Time (sec)	Ratio	FMA (M)	Time (sec)	Ratio	FMA (M)
0	inf	0.0080	0	inf	0.0105	0	inf	0.0094
92	15.585	0.0076	90	7.76	0.0093	90	7.265	0.0082
231	4.1	0.0065	324	2.115	0.0071	275	2.88	0.0070
358	2.64	0.0058	551	1.215	0.0058	440	1.755	0.0060
489	1.685	0.0050	781	0.685	0.0043	609	1.035	0.0048
715	1.14	0.0043	1238	0.395	0.0030	919	0.66	0.0037
943	0.86	0.0037	1668	0.27	0.0022	1229	0.465	0.0030
1171	0.66	0.0032	2099	0.18	0.0016	1537	0.37	0.0025
1397	0.55	0.0029	2528	0.13	0.0012	1845	0.315	0.0022
1626	0.45	0.0025	2958	0.105	0.0010	2154	0.215	0.0017
2060	0.315	0.0019	3388	0.085	0.0008	2462	0.15	0.0012
2490	0.235	0.0015	-	-	-	-	-	-

Table S15. Ratio of NMR integrated peak areas and concentration of the adduct as a function of time at 108°C for the furan - N-methyl maleimide adduct in TCE. Two trials are present.

108°C					
Time (sec)	Ratio	FMA (M)	Time (sec)	Ratio	FMA (M)
0	inf	0.0080	0	inf	0.0105
75	16.28	0.0076	90	5.66	0.0089
211	3.025	0.0060	326	1.625	0.0065
337	1.69	0.0051	552	0.895	0.0050
462	1.195	0.0044	781	0.58	0.0039
588	0.88	0.0038	1013	0.355	0.0027
717	0.635	0.0031	1444	0.21	0.0018
944	0.515	0.0027	1872	0.14	0.0013
1171	0.325	0.0020	2300	0.105	0.0010
1399	0.22	0.0014	2730	0.065	0.0006
1636	0.175	0.0012	-	-	-

Table S16. Ratio of NMR integrated peak areas and concentration of the adduct as a function of time at 110°C for the furan - N-methyl maleimide adduct in TCE. Three trials are present.

110°C								
Time (sec)	Ratio	FMA (M)	Time (sec)	Ratio	FMA (M)	Time (sec)	Ratio	FMA (M)
0	inf	0.0080	0	inf	0.0094	0	inf	0.0105
70	11.535	0.0074	90	9.885	0.0085	90	8.23	0.0094
209	2.625	0.0058	190	3.325	0.0072	224	2.475	0.0075
336	1.44	0.0047	287	1.535	0.0057	352	1.16	0.0056
462	0.995	0.0040	452	0.895	0.0044	580	0.635	0.0041
586	0.72	0.0034	617	0.595	0.0035	810	0.385	0.0029
712	0.6	0.0030	782	0.44	0.0029	1038	0.225	0.0019
841	0.47	0.0026	955	0.345	0.0024	1468	0.125	0.0012
1068	0.355	0.0021	1123	0.21	0.0016	1955	0.08	0.0008
1296	0.235	0.0015	1444	0.125	0.0010	-	-	-

Table S17. Ratio of NMR integrated peak areas and concentration of the adduct as a function of time at 112°C and 114°C for the furan - N-methyl maleimide adduct in TCE. A total of three trials are present.

112°C			114°C					
Time (sec)	Ratio	FMA (M)	Time (sec)	Ratio	FMA (M)	Time (sec)	Ratio	FMA (M)
0	inf	0.0080	0	inf	0.0094	0	inf	0.0105
143	4.94	0.0067	75	15.785	0.0088	60	21.33	0.0100
220	1.97	0.0053	140	3.84	0.0074	146	3.095	0.0079
347	1.055	0.0041	197	2.11	0.0064	221	1.545	0.0064
474	0.715	0.0034	256	1.265	0.0052	300	0.855	0.0048
602	0.565	0.0029	349	0.815	0.0042	425	0.5	0.0035
728	0.36	0.0021	442	0.585	0.0035	552	0.335	0.0026
857	0.26	0.0017	535	0.425	0.0028	677	0.24	0.0020
-	-	-	631	0.295	0.0021	804	0.16	0.0014
-	-	-	802	0.225	0.0017	932	0.105	0.0010
-	-	-	967	0.125	0.0010	1161	0.05	0.0005

Table S18. Ratio of NMR integrated peak areas and concentration of the adduct as a function of time at 116°C for the furan - N-methyl maleimide adduct in TCE. A total of one trial is present.

116°C		
Time (sec)	Ratio	FMA (M)
0	inf	0.0080
60	14.975	0.0075
154	2.075	0.0054
230	1.105	0.0042
306	0.755	0.0035
384	0.545	0.0028
466	0.33	0.0020
599	0.18	0.0012

Table S19. Ratio of NMR integrated peak areas and concentration of the adduct as a function of time at 118°C for the furan - N-methyl maleimide adduct in TCE. A total of three trials are present.

118°C								
Time (sec)	Ratio	FMA (M)	Time (sec)	Ratio	FMA (M)	Time (sec)	Ratio	FMA (M)
0	inf	0.0080	0	inf	0.0094	0	inf	0.0105
132	1.825	0.0052	70	9.015	0.0084	70	9.495	0.0095
207	0.94	0.0039	151	1.98	0.0062	153	1.975	0.0070
284	0.575	0.0029	208	1.04	0.0048	228	0.915	0.0050
360	0.36	0.0021	275	0.615	0.0036	302	0.54	0.0037
436	0.235	0.0015	331	0.455	0.0029	377	0.37	0.0028
-	-	-	389	0.31	0.0022	452	0.275	0.0023
-	-	-	445	0.29	0.0021	526	0.18	0.0016
-	-	-	501	0.205	0.0016	605	0.13	0.0012
-	-	-	564	0.155	0.0013	-	-	-
-	-	-	619	0.12	0.0010	-	-	-

Furan- n-methyl maleimide in DMSO

Table S20. Ratio of NMR integrated peak areas and concentration of the adduct as a function of time at 105°C for the furan - N-methyl maleimide adduct in DMSO. A total of three trials are present.

105°C								
Time (sec)	Ratio	FMA (M)	Time (sec)	Ratio	FMA (M)	Time (sec)	Ratio	FMA (M)
0	inf	0.0090	0	inf	0.0098	0	inf	0.0095
67	22.57	0.0087	90	12.7	0.0091	90	14.805	0.0089
300	4.22	0.0073	329	2.665	0.0071	329	2.61	0.0068
533	1.735	0.0057	761	1.18	0.0053	759	1.14	0.0050
960	0.93	0.0044	1190	0.705	0.0041	1190	0.665	0.0038
1389	0.59	0.0034	1620	0.46	0.0031	1623	0.435	0.0029
1818	0.37	0.0024	2049	0.33	0.0024	2054	0.305	0.0022
2248	0.27	0.0019	2479	0.23	0.0018	2484	0.2	0.0016
2675	0.175	0.0013	2907	0.17	0.0014	2916	0.14	0.0012
3105	0.17	0.0013	-	-	-	3347	0.105	0.0009

Table S21. Ratio of NMR integrated peak areas and concentration of the adduct as a function of time at 109°C for the furan - N-methyl maleimide adduct in DMSO. A total of three trials are present.

108°C								
Time (sec)	Ratio	FMA (M)	Time (sec)	Ratio	FMA (M)	Time (sec)	Ratio	FMA (M)
0	inf	0.0090	0	inf	0.0098	0	inf	0.0095
60	11.49	0.0083	90	9.035	0.0088	90	10.895	0.0087
294	2.745	0.0066	327	2.34	0.0069	322	2.285	0.0066
530	1.43	0.0053	554	1.215	0.0054	551	1.195	0.0051
756	0.75	0.0039	784	0.675	0.0040	783	0.685	0.0038
1185	0.4	0.0026	1213	0.375	0.0027	1214	0.38	0.0026
1613	0.235	0.0017	1645	0.225	0.0018	1645	0.22	0.0017
2041	0.165	0.0013	2073	0.175	0.0015	2080	0.14	0.0012
2468	0.095	0.0008	2503	0.105	0.0009	2510	0.07	0.0006
2899	0.075	0.0006	2931	0.065	0.0006	-	-	-

Table S22. Ratio of NMR integrated peak areas and concentration of the adduct as a function of time at 110°C for the furan - N-methyl maleimide adduct in DMSO. A total of two trials are present.

110°C					
Time (sec)	Ratio	FMA (M)	Time (sec)	Ratio	FMA (M)
0	inf	0.0095	0	inf	0.0106
26	21.205	0.0091	60	8.425	0.0095
258	2.16	0.0065	294	1.66	0.0066
483	1.03	0.0048	527	0.67	0.0043
709	0.62	0.0036	959	0.29	0.0024
963	0.42	0.0028	1389	0.16	0.0015
1194	0.26	0.0020	1817	0.105	0.0010
1431	0.18	0.0014	2247	0.07	0.0007
1860	0.09	0.0008	2673	0.095	0.0009
2292	0.001	0.0000	3105	0.045	0.0005

Table S23. Ratio of NMR integrated peak areas and concentration of the adduct as a function of time at 111°C for the furan - N-methyl maleimide adduct in DMSO. A total of three trials are present.

111°C								
Time (sec)	Ratio	FMA (M)	Time (sec)	Ratio	FMA (M)	Time (sec)	Ratio	FMA (M)
0	inf	0.0090	0	inf	0.0098	0	inf	0.0095
60	11.195	0.0083	90	6.295	0.0085	90	5.65	0.0080
298	1.805	0.0058	325	1.41	0.0057	323	1.4	0.0055
525	0.88	0.0042	552	0.73	0.0041	550	0.725	0.0040
750	0.53	0.0031	778	0.445	0.0030	778	0.455	0.0030
980	0.315	0.0022	1007	0.305	0.0023	1005	0.31	0.0022
1205	0.21	0.0016	1234	0.205	0.0017	1231	0.18	0.0014
1438	0.13	0.0010	1460	0.14	0.0012	1456	0.125	0.0011
1868	0.08	0.0007	1691	0.105	0.0009	1692	0.075	0.0007
2308	0.055	0.0005	-	-	-	-	-	-

Table S24. Ratio of NMR integrated peak areas and concentration of the adduct as a function of time at 112°C for the furan - N-methyl maleimide adduct in DMSO. A total of one trials is present.

112°C		
Time (sec)	Ratio	FMA (M)
0	inf	0.0106
60	6.9	0.0093
294	1.26	0.0059
521	0.555	0.0038
750	0.355	0.0028
982	0.18	0.0016
1415	0.115	0.0011
1903	0.075	0.0007
2272	0.035	0.0004

Table S25. Ratio of NMR integrated peak areas and concentration of the adduct as a function of time at 114°C for the furan - N-methyl maleimide adduct in DMSO. A total of four trials are present.

114°C											
Time (sec)	Ratio	FMA (M)	Time (sec)	Ratio	FMA (M)	Time (sec)	Ratio	FMA (M)	Time (sec)	Ratio	FMA (M)
0	inf	0.0090	0	inf	0.0106	0	inf	0.0098	0	inf	0.0095
67	23.14	0.0087	60	11.405	0.0097	90	8.455	0.0088	90	12.655	0.0088
201	2.605	0.0065	192	2.02	0.0071	226	1.92	0.0065	244	2.16	0.0065
328	1.22	0.0050	318	0.99	0.0053	352	1.055	0.0050	480	0.58	0.0035
456	0.61	0.0034	449	0.53	0.0037	489	0.525	0.0034	708	0.305	0.0022
682	0.33	0.0022	675	0.26	0.0022	715	0.285	0.0022	838	0.2	0.0016
910	0.16	0.0012	902	0.16	0.0015	945	0.165	0.0014	1066	0.13	0.0011
1144	0.1	0.0008	1129	0.105	0.0010	1172	0.105	0.0009	1300	0.06	0.0005
1585	0.06	0.0005	1355	0.075	0.0007	1404	0.06	0.0006	1745	0.045	0.0004
-	-	-	-	-	-	1846	0.03	0.0003	-	-	-

Table S26. Ratio of NMR integrated peak areas and concentration of the adduct as a function of time at 115°C and 116°C for the furan - N-methyl maleimide adduct in DMSO. A total of two trials are present.

115°C			116°C		
Time (sec)	Ratio	FMA (M)	Time (sec)	Ratio	FMA (M)
0	inf	0.0095	0	inf	0.0106
155	3.84	0.0075	74	13.14	0.0099
279	1.44	0.0056	156	3.005	0.0080
403	0.765	0.0041	237	1.18	0.0057
529	0.525	0.0033	366	0.58	0.0039
654	0.32	0.0023	491	0.33	0.0026
779	0.245	0.0019	624	0.215	0.0019
904	0.175	0.0014	850	0.115	0.0011
1038	0.125	0.0011	1080	0.075	0.0007
1267	0.09	0.0008	-	-	-
1502	0.01	0.0001	-	-	-

Table S27. Ratio of NMR integrated peak areas and concentration of the adduct as a function of time at 117°C for the furan - N-methyl maleimide adduct in DMSO. A total of three trials are present.

117°C								
Time (sec)	Ratio	FMA (M)	Time (sec)	Ratio	FMA (M)	Time (sec)	Ratio	FMA (M)
0	inf	0.0090	0	inf	0.0098	0	inf	0.0095
143	4.48	0.0074	75	18.71	0.0093	90	12.9	0.0088
221	1.45	0.0054	159	3.065	0.0074	172	3.115	0.0072
345	0.72	0.0038	239	1.14	0.0052	252	1.145	0.0050
469	0.42	0.0027	365	0.575	0.0036	377	0.6	0.0035
593	0.27	0.0019	491	0.32	0.0024	502	0.37	0.0026
721	0.14	0.0011	619	0.19	0.0016	627	0.195	0.0015
964	0.07	0.0006	744	0.13	0.0011	752	0.13	0.0011
-	-	-	873	0.07	0.0006	880	0.085	0.0007

Table S28. Ratio of NMR integrated peak areas and concentration of the adduct as a function of time at 118°C and 119°C for the furan - N-methyl maleimide adduct in DMSO. A total of two trials are present.

118°C			119°C		
Time (sec)	Ratio	FMA (M)	Time (sec)	Ratio	FMA (M)
0	inf	0.0106	0	inf	0.0095
60	13.68	0.0099	110	6.17	0.0082
142	2.48	0.0076	184	1.27	0.0053
294	0.475	0.0034	260	1.04	0.0048
420	0.27	0.0023	335	0.59	0.0035
545	0.155	0.0014	409	0.375	0.0026
670	0.11	0.0011	485	0.305	0.0022
976	0.07	0.0007	564	0.175	0.0014
-	-	-	692	0.125	0.0011
-	-	-	818	0.01	0.0001

Table S29. Ratio of NMR integrated peak areas and concentration of the adduct as a function of time at 120°C for the furan - N-methyl maleimide adduct in DMSO. A total of four trials are present.

120°C											
Time (sec)	Ratio	FMA (M)	Time (sec)	Ratio	FMA (M)	Time (sec)	Ratio	FMA (M)	Time (sec)	Ratio	FMA (M)
0	inf	0.0090	0	inf	0.0106	0	inf	0.0098	0	inf	0.0095
131	3.755	0.0071	60	15.6	0.0100	60	32.675	0.0095	80	12.995	0.0088
207	1.29	0.0051	149	1.635	0.0066	145	2.61	0.0071	165	1.815	0.0061
281	0.685	0.0037	223	0.65	0.0042	218	1.04	0.0050	240	0.825	0.0043
356	0.375	0.0025	298	0.36	0.0028	296	0.51	0.0033	315	0.425	0.0028
429	0.21	0.0016	372	0.225	0.0019	371	0.32	0.0024	389	0.285	0.0021
509	0.12	0.0010	446	0.175	0.0016	447	0.19	0.0016	465	0.16	0.0013
633	0.05	0.0004	521	0.095	0.0009	526	0.13	0.0011	542	0.105	0.0009
-	-	-	597	0.07	0.0007	-	-	-	-	-	-

Table S30. Ratio of NMR integrated peak areas and concentration of the adduct as a function of time at 122°C and 125°C for the furan - N-methyl maleimide adduct in DMSO. A total of two trials are present.

122°C			125°C		
Time (sec)	Ratio	FMA (M)	Time (sec)	Ratio	FMA (M)
0	inf	0.0095	0	inf	0.0095
108	4.83	0.0079	110	3.06	0.0072
183	1.47	0.0056	187	0.785	0.0042
259	0.67	0.0038	262	0.335	0.0024
333	0.385	0.0026	338	0.155	0.0013
409	0.23	0.0018	414	0	0.0000
484	0.15	0.0012	-	-	-
558	0.105	0.0009	-	-	-
634	0.01	0.0001	-	-	-

Calculated rate constants:
Rate constant units: s^{-1}

Furan-maleic anhydride - TCE

Table S31. The rate constants for the reverse Diels-Alder reaction obtained at each temperature for the furan-maleic anhydride adduct in TCE. The rate constants are obtained from fits in DynaFit 4, as described in the main text. Also given are the standard errors obtained from the fits.

T (K)	k	+/-	k	+/-	k	+/-
333	0.0707	0.0009	0.0686	0.0009	0.0744	0.0017
338	0.1210	0.0028	0.1340	0.0052	0.1403	0.0067
343	0.2460	0.0058	0.2447	0.0027	0.2330	0.0020
348	0.3799	0.0177	0.4514	0.0128	0.4846	0.0329
353	0.7522	0.0479	0.7402	0.0426	0.7446	0.0430
358	0.9056	0.1051	1.1885	0.1215	1.3293	0.1998

Furan-maleic anhydride - acetonitrile

Table S32. The rate constants for the reverse Diels-Alder reaction obtained at each temperature for the furan-maleic anhydride adduct in ACN. The rate constants are obtained from fits in DynaFit 4, as described in the main text. Also given are the standard errors obtained from the fits.

T (K)	k	+-	k	+-	k	+-
333	0.0387	0.0006	0.0357	0.0006	0.0371	0.0005
338	0.0804	0.0013	0.0771	0.0012	0.0752	0.0023
343	0.1252	0.0026	0.1396	0.0026	0.1536	0.0020
346	0.1907	0.0070	0.1906	0.0052	0.2224	0.0027
349	0.2352	0.0167	0.2488	0.0071	0.3410	0.0067
352	0.3496	0.0193	0.3499	0.0122	0.5598	0.0215

Furan- n-methyl maleimide - TCE

Table S33. The rate constants for the reverse Diels-Alder reaction obtained at each temperature for the furan - N-methyl maleimide adduct in TCE. The rate constants are obtained from fits in DynaFit 4, as described in the main text. Also given are the standard errors obtained from the fits.

T (K)	k	+-	T (K)	k	+-	T (K)	k	+-
378	0.0399	0.0014	375	0.0287	0.0013	379	0.0456	0.0021
381	0.0704	0.0017	379	0.0468	0.0018	381	0.0603	0.0025
383	0.0757	0.0026	383	0.0910	0.0022	383	0.0832	0.0039
385	0.1104	0.0035	387	0.1380	0.0035	387	0.1570	0.0027
389	0.1897	0.0069	391	0.2233	0.0055	391	0.2225	0.0056
391	0.2284	0.0062	-	-	-	-	-	-

Furan - n-methyl maleimide - DMSO

Table S34. The rate constants for the reverse Diels-Alder reaction obtained at each temperature for the furan - N-methyl maleimide adduct in TCE. The rate constants are obtained from fits in DynaFit 4, as described in the main text. Also given are the standard errors obtained from the fits.

T (K)	k	+-	T (K)	k	+-	k	+-	k	+-	T (K)	k	+-
383	0.0789	0.0011	378	0.0394	0.0014	0.0393	0.0009	0.0418	0.0007	383	0.0589	0.0054
388	0.1246	0.0032	381	0.0564	0.0015	0.0558	0.0015	0.0629	0.0013	385	0.0857	0.0047
392	0.2012	0.0086	384	0.0803	0.0027	0.0835	0.0014	0.0914	0.0019	387	0.1226	0.0046
395	0.2697	0.0128	387	0.1175	0.0057	0.1189	0.0027	0.1186	0.0066	389	0.1578	0.0062
398	0.3645	0.0440	390	0.1764	0.0059	0.1908	0.0053	0.1825	0.0054	391	0.1852	0.0164
-	-	-	393	0.2979	0.0217	0.2627	0.0130	0.2744	0.0144	393	0.2884	0.0092

5. Extracted kinetic parameters

Table S35. Values (and associated errors) of all kinetic parameters obtained from the fits shown in Figure 4

**Adduct:Solvent**	ν_N /s^{-1}	E_A /$\mathrm{kJ \cdot mol^{-1}}$	$\Delta H^{\dagger}$ /$\mathrm{kJ \cdot mol^{-1}}$	$\Delta S^{\dagger}$ /$\mathrm{J \cdot mol^{-1}}$
F-MA:TCE	$(2.90 \pm 4) \cdot 10^{13}$	104 ± 1	102 ± 3	178 ± 7
F-MA:ACN	$(4.23 \pm 5) \cdot 10^{14}$	114 ± 1	111 ± 2	200 ± 6
F-MM:TCE	$(6.73 \pm 2) \cdot 10^{14}$	129 ± 4	126 ± 8	203 ± 18
F-MM:DMSO	$(3.34 \pm 1) \cdot 10^{15}$	134 ± 4	132 ± 7	215 ± 17

6. References

(1) Anderson, W. K.; Milowsky, A. S. *J. Org. Chem.* 1985, *50* (25), 5423–5424.
